# Efficacy of Flecainide in Catecholaminergic Polymorphic Ventricular Tachycardia Is Mutation-Independent but Reduced by Calcium Overload

**DOI:** 10.3389/fphys.2019.00992

**Published:** 2019-08-13

**Authors:** Hyun Seok Hwang, Marcelo P. Baldo, Jose Pindado Rodriguez, Michela Faggioni, Bjorn C. Knollmann

**Affiliations:** ^1^Department of Nutrition, Food and Exercise Sciences, Florida State University, Tallahassee, FL, United States; ^2^Division of Clinical Pharmacology, Oates Institute for Experimental Therapeutics, Vanderbilt University School of Medicine, Nashville, TN, United States

**Keywords:** catecholaminergic polymorphic ventricular tachycardia, flecainide, calcium overload, calsequestrin (Casq2), cardiac ryanodine receptor (RyR2)

## Abstract

**Background:**

The dual Na^+^ and cardiac Ca^2+^-release channel inhibitor, Flecainide (FLEC) is effective in patients with catecholaminergic polymorphic ventricular tachycardia (CPVT), a disease caused by mutations in cardiac Ca^2+^-release channels (RyR2), calsequestrin (Casq2), or calmodulin. FLEC suppresses spontaneous Ca^2+^ waves in Casq2-knockout (Casq2^−/−^) cardiomyocytes, a CPVT model. However, a report failed to find FLEC efficacy against Ca^2+^ waves in another CPVT model, RyR2-R4496C heterozygous mice (RyR2^R4496C+/−^), raising the possibility that FLEC efficacy may be mutation dependent.

**Objective:**

To address this controversy, we compared FLEC in Casq2^−/−^ and RyR2^R4496C+/−^ cardiomyocytes and mice under identical conditions.

**Methods:**

After 30 min exposure to FLEC (6 μM) or vehicle (VEH), spontaneous Ca^2+^ waves were quantified during a 40 s pause after 1 Hz pacing train in the presence of isoproterenol (ISO, 1 μM). FLEC efficacy was also tested *in vivo* using a low dose (LOW: 3 mg/kg ISO + 60 mg/kg caffeine) or a high dose catecholamine challenge (HIGH: 3 mg/kg ISO + 120 mg/kg caffeine).

**Results:**

In cardiomyocytes, FLEC efficacy was dependent on extracellular [Ca^2+^]. At 2 mM [Ca^2+^], only Casq2^−/−^ myocytes exhibited Ca^2+^ waves, which were strongly suppressed by FLEC. At 3 mM [Ca^2+^] both groups exhibited Ca^2+^ waves that were suppressed by FLEC. At 4 mM [Ca^2+^], FLEC no longer suppressed Ca^2+^ waves in both groups. Analogous to the results in myocytes, RyR2^R4496C+/−^ mice (*n* = 12) had significantly lower arrhythmia scores than Casq2^−/−^ mice (*n* = 9), but the pattern of FLEC efficacy was similar in both groups (i.e., reduced FLEC efficacy after HIGH dose catecholamine challenge).

**Conclusion:**

FLEC inhibits Ca^2+^ waves in RyR2^R4496C+/−^ cardiomyocytes, indicating that RyR2 channel block by FLEC is not mutation-specific. However, FLEC efficacy is reduced by Ca^2+^ overload *in vitro* or by high dose catecholamine challenge *in vivo*, which could explain conflicting literature reports.

## Introduction

Catecholaminergic polymorphic ventricular tachycardia (CPVT) is an inherited arrhythmogenic syndrome characterized by polymorphic ventricular tachycardia in response to exercise or emotional stress, leading sometimes to sudden cardiac death (Huikuri et al., 2001). In approximately 60% of cases, CPVT is caused by mutations in three genes encoding sarcoplasmic reticulum (SR) Ca^2+^ handling proteins, the cardiac ryanodine receptor (RyR2), calsequestrin 2 (Casq2) (Faggioni et al., 2012), or calmodulin (CaM) (Nyegaard et al., 2012; Crotti et al., 2013). At the cellular level, defective SR Ca^2+^ handling is characterized by abnormal Ca^2+^ leak that generates spontaneous Ca^2+^ waves, triggering delayed afterdepolarization (DAD) and ventricular tachycardia (Chopra et al., 2009; MacLennan and Chen, 2009; Faggioni and Knollmann, 2012; Hwang et al., 2014; Gomez-Hurtado et al., 2016). Drug therapy includes ß-blockers and non-dihydropyridine Ca^2+^ channel blockers. Left cardiac sympathectomy has been used with relative success in drug-refractory patients, but it is not completely effective. Although implantable cardioverter defibrillators (ICD) are recommended as therapy to prevent sudden deaths, the painful shocks, increased stress, and pro-arrhythmic liability after shock delivery reduce their effectiveness (Pflaumer and Davis, 2012).

Another therapeutic option is the class Ic anti-arrhythmic drug flecainide, which has clinical efficacy for preventing ventricular arrhythmias in CPVT patients (Biernacka and Hoffman, 2011; Pott et al., 2011; van der Werf et al., 2011). We first showed that flecainide decreased arrhythmias in two drug-refractory patients and also in Casq2-knockout (Casq2^−/−^) mice, a CPVT model (Watanabe et al., 2009). In addition to its well-known Na^+^ channel blocking properties, block of RyR2 channels likely contributes to flecainide action in CPVT. Using Casq2^−/−^ myocytes as a model system, the reduction in Ca^2+^ spark mass and the associated blockage of the spontaneous Ca^2+^ waves were attributed to blocking the RyR2 in its open state (Watanabe et al., 2009; Hilliard et al., 2010). However, a study by Liu et al. (2011) failed to find flecainide efficacy against Ca^2+^ waves in another CPVT model, the RyR2^R4496C+/–^ mice, and suggested that the antiarrhythmic effect of flecainide was only the result of its Na^+^ channel blocking properties (Liu et al., 2011). These conflicting results raise the possibility that flecainide efficacy against RyR2-mediated Ca^2+^ waves may be dependent on a specific genetic mutation. To address this controversy, we compared flecainide efficacy in Casq2^−/−^ and RyR2^R4496C+/–^ cardiac myocytes and mice under identical conditions.

## Materials and Methods

### Experimental Procedures and Animals

Adult Casq2^−/−^ and RyR2^R4496C+/–^ mutant mice at the age between 12 and 36 weeks old were used for all experiments. Animals were housed with a maximum of five animals per cages. Animals had free access to food and water. All compounds such as flecainide, isoproterenol, caffeine etc., were dissolved in pure water. The vehicle group consisted of the same amount of pure water. All protocols followed the Guide for the Care and Use of Laboratory Animals (NIH publication No. 85-23, revised 1996) and were approved by the institutional Committee of Ethics in Animal Research at Vanderbilt University Medical Center.

### Surface ECG

Animals were anesthetized with 1.5% isoflurane with 100% O_2_ and placed on a heating pad. Loss of toe-pinch reflex and respiration rate was used to monitor levels of anesthesia (Watanabe et al., 2009; Hwang et al., 2011). Baseline electrocardiogram (ECG) was recorded for 5 min, followed by an additional 10 min after intraperitoneal administration of the ß-adrenergic receptor agonist isoproterenol (3 mg/kg) plus low-dose caffeine (LOW, 60 mg/kg), or isoproterenol (3 mg/kg) plus high dose caffeine (HIGH, 120 mg/kg). Flecainide (20 mg/kg) was administered 15 min before isoproterenol challenge. The experiments were conducted in a randomized crossover design, with each mouse receiving vehicle or flecainide plus catecholamine challenge. Experiments were conducted at an interval of 72 h to minimize carryover effects of previous pharmacological interventions (Watanabe et al., 2009).

### Cell Isolation and Ca^2+^ Fluorescence Recordings

Ventricular myocytes were isolated by a modified collagenase/protease method as previously described (Knollmann et al., 2000, 2003, 2006; Hwang et al., 2011). All experiments were conducted in Tyrode’s solution containing (in mM): CaCl_2_ 2, 3, or 4, NaCl 134, KCl 5.4, MgCl_2_ 1, glucose 10, and HEPES 10, pH adjusted to 7.4 with NaOH. Fura-2 AM (Molecular Probes Inc., Eugene, Ore)-loaded cardiac myocytes were field-stimulated at 1 Hz for 20 s to reach a constant Ca^2+^ transient height. All single cell experiments were conducted at room temperature. After 30 min exposure of Fura-2 AM loaded, quiescent cardiomyocytes to FLEC (6 μM) or vehicle (VEH), spontaneous Ca^2+^ waves were quantified during a 40 s pause after the 1 Hz pacing train in the presence of isoproterenol (ISO, 1 μM). At the end of the pause, myocytes were exposed to 10 mM caffeine for 5 s using a rapid concentration clamp system. SR Ca^2+^ content was estimated by the amplitude of the Ca^2+^ transient induced by rapid Caffeine application. Spontaneous SR Ca^2+^ waves (SCWs) were defined as any spontaneous increase of 0.07 ratiometric units or more from the diastolic *F*_ratio_. To calculate incidence of spontaneous Ca^2+^ releases (SCRs), cells that displayed a specific event (spontaneous Ca^2+^ wave, triggered beat) during the 40 s pause period were counted as positive, and then expressed as a percentage of total cells analyzed. To estimate the inhibitory concentration at which flecainide achieved 50% of the maximal effect (IC_50_) of Ca wave suppression, we used 0 and 1 as asymptotes for maximum and minimum inhibition and fitted the mean and SEM of three flecainide concentration data sets (0, 2, 6 uM) to a Boltzmann Function using the OriginPro 9.0 software.

### Statistical Analysis

Differences among the groups were assessed using one-way ANOVA. The incidence of arrhythmia and spontaneous Ca^2+^ waves were compared by non-parametric Mann–Whitney *U* test. Results were considered statistically significant if the *p*-value was less than 0.05.

## Results

### Anti-arrhythmic Efficacy of Flecainide in Cardiomyocytes From Different CPVT Models

**FIGURE 1:**
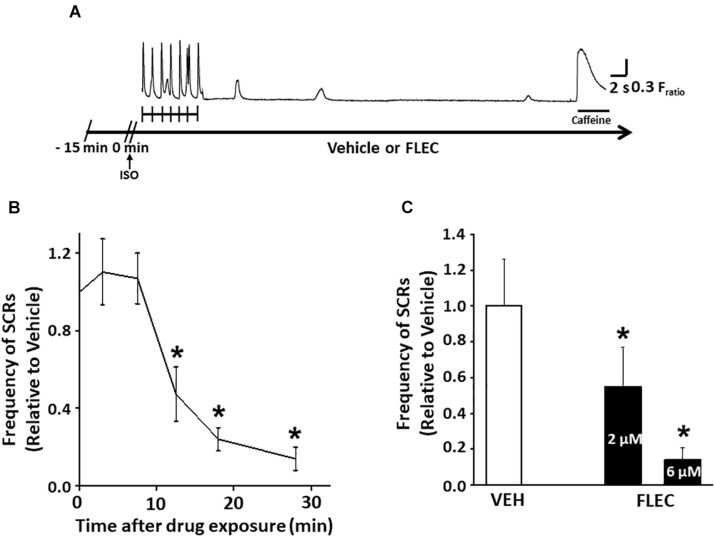
Flecainide inhibition of spontaneous Ca releases (SCRs) exhibited a significant lag time in intact cardiomyocytes. **(A)** Experimental protocol in field-stimulated myocytes. **(B)** Exposure time-dependent effects of flecainide (FLEC). **(C)** Concentration-dependent effects of flecainide on the rate of Ca^2+^ waves after acute isoproterenol exposure (1 μM) in intact myocytes. IC_50_ values (mean ± SEM) were obtained by fitting the values to a Boltzmann function. ^*^p < 0.05 vs. VEH. *n* = 22–33 myocytes per group.

To address the controversy regarding the possible mutation-dependent efficacy of flecainide on Ca^2+^ waves (Hilliard et al., 2010; Liu et al., 2011), we chose two widely published mouse models of CPVT: mice lacking calsequestrin (Casq2^−/−^ mice) (Knollmann et al., 2006) and mice heterozygous for the RyR2-R4496C mutation (RyR2^R4496C+/–^ mice) (Cerrone et al., 2005). Single cardiomyocytes of both CPVT models exhibit spontaneous Ca^2+^ waves due to increased RyR2 activity at diastolic [Ca^2+^] (Knollmann et al., 2006; Liu et al., 2006). To ensure optimal experimental conditions, we first established the time-dependence of Ca^2+^ wave suppression by flecainide in single Casq2^−/−^ cardiomyocytes (Figure 1). Spontaneous Ca^2+^ releases (SCRs) events were defined as either spontaneous Ca^2+^ waves or spontaneously triggered beats. SCRs were elicited by applying a pacing train in the presence of isoproterenol (Figure 1A). Suppression of SCR events by flecainide was not observed until after 10 min extracellular incubation, with maximum SCR suppression achieved after 30 min (Figure 1B). We then established the concentration dependence of flecainide using the 30 min incubation of time point. Based on testing three flecainide concentrations (0, 2, and 6 μM), the flecainide IC_50_ was approximately 2.0 ± 0.2 μM (Figure 1C). Higher concentration could not be tested because Flecainide’s Na^+^ channel block renders cardiomyocytes unpacable.

**FIGURE 2:**
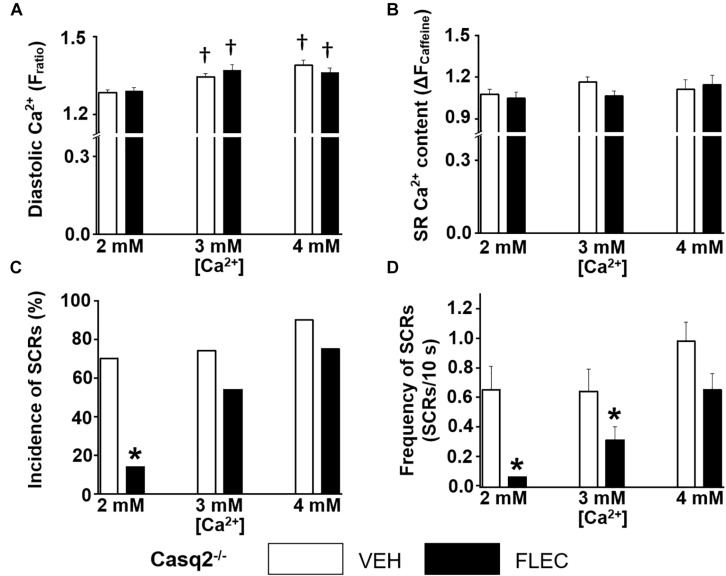
Effect of flecainide on intracellular Ca^2+^ handling of cardiomyocytes isolated from Casq2^−/−^ mice. **(A)** Diastolic intracellular Ca^2+^. **(B)** The amplitude of caffeine-induced Ca^2+^ transients (Δ*F*_ratio_) was used as estimates of sarcoplasmic reticulum (SR) Ca^2+^ content. Incidence **(C)** and frequency **(D)** of SCRs. The incidence of SCRs was analyzed by Fisher exact test, and the frequency of SCRs was compared by non-parametric Mann–Whitney *U* test. †*p* < 0.05 vs. 2 mM [Ca^2+^] VEH, ^*^p < 0.05 vs. each VEH, *n* = 20–33 myocytes per group.

**FIGURE 3:**
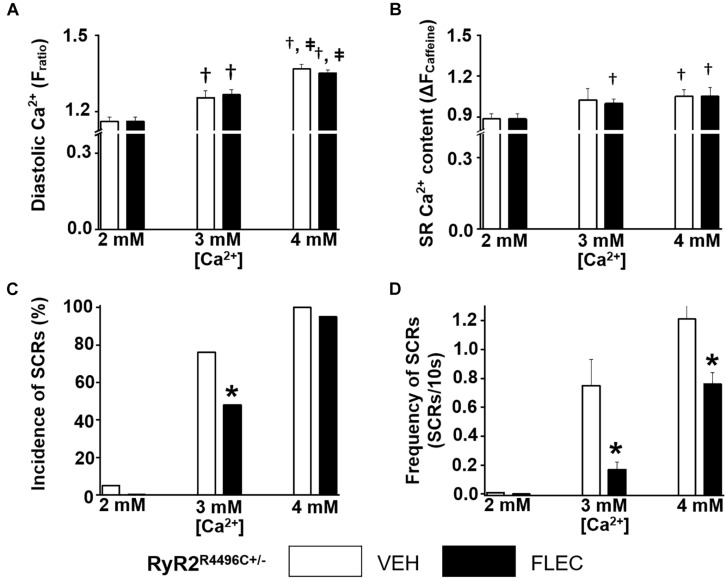
Effect of flecainide on intracellular Ca^2+^ handling of cardiomyocytes isolated from RyR2^R4496C+/–^ mice. **(A)** Diastolic intracellular Ca^2+^. **(B)** The amplitude of caffeine-induced Ca^2+^ transients (Δ*F*_ratio_) was used as estimates of sarcoplasmic reticulum (SR) Ca^2+^ content. Incidence **(C)** and frequency **(D)** of SCRs. The incidence of SCRs was analyzed by Fisher exact test and the frequency of SCRs was compared by non-parametric Mann–Whitney *U* test. †*p* < 0.05 vs. 2 mM [Ca^2+^] VEH, ‡ *p* < 0.05 vs. 3 mM [Ca^2+^] VEH, ^*^p < 0.05 vs. each VEH, *n* = 20–33 myocytes per group.

Next, we compared the rate and incidence of spontaneous Ca^2+^ waves of ventricular cardiomyocytes isolated from Casq2^−/−^ and RyR2^R4496C+/–^ mice under identical experimental conditions (Figures 2, 3). At the customary extracellular [Ca^2+^] of 2 mM, the rate of Ca^2+^ waves was significantly higher in Casq2^−/−^ compared to RyR2^R4496C+/–^ cardiomyocytes (SCRs/10s, 0.65 ± 0.16 vs. 0.05 ± 0.01, Figures 2, 3). This result suggests that the molecular Ca^2+^ handling defect caused by the RyR2 mutation is less severe than that caused by loss of calsequestrin. Flecainide significantly reduced the incidence of Ca^2+^ waves in Casq2^−/−^ cardiomyocytes (70% vs. 14%, Figure 2C), and completely prevented Ca^2+^ waves in RyR2^R4496C+/–^ cardiomyocytes (5% vs. 0%, Figure 3C).

### Cardiomyocyte Ca^2+^ Overload Reduces Flecainide Efficacy

Since in our experiments flecainide suppressed Ca^2+^ waves equally in Casq2^−/−^ and RyR2^R4496C+/–^ cardiomyocytes (Figures 2, 3), we reasoned that differences in experimental conditions may explain the reported lack of flecainide efficacy against Ca^2+^ waves in RyR2^R4496C+/–^ cardiomyocytes (Liu et al., 2011). In order to test this hypothesis, we exposed cardiomyocytes to increasing extracellular [Ca^2+^]. Raising extracellular [Ca^2+^] to 3 and 4 mM significantly increased intracellular diastolic [Ca^2+^] (Figures 2A, 3A), resulting in higher rates of SCRs in both models (Figures 2C,D, 3C,D). Consistent with its reported lack of effect on SR Ca^2+^ leak (Hilliard et al., 2010), flecainide did not have a significant effect on diastolic [Ca^2+^] at all extracellular [Ca^2+^] tested. Although still effective at 3 mM [Ca^2+^], flecainide’s ability to inhibit spontaneous Ca^2+^ waves was severely blunted at the highest [Ca^2+^] tested. Taken together, these results indicate that flecainide’s efficacy against Ca^2+^ waves is reduced by cellular Ca^2+^ overload, regardless of the CPVT mutation.

### *In vivo* Anti-arrhythmic Efficacy of Flecainide in Different CPVT Models

**FIGURE 4:**
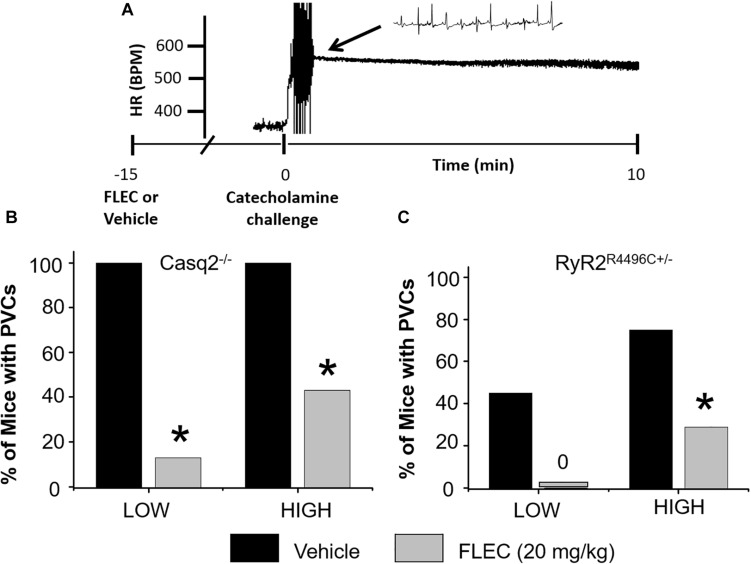
Anti-arrhythmic efficacy of flecainide *in vivo*. **(A)** Representative example of heart rate (HR) recorded from an anesthetized Casq2^−/−^ mouse during a catecholamine stress challenge. Summary data of ventricular premature complexes (PVCs) for Casq2^−/−^ **(B)** and RyR2^R4496C+/–^ **(C)** mice. Flecainide: 20 mg/kg (15 min pretreatment), LOW: ISO (3 mg/kg) + Caff (60 mg/kg), HIGH: ISO (3 mg/kg) + Caff (120 mg/kg). ^*^p < 0.05 vs. VEH, *n* = 9–12 mice per group.

To elicit CPVT *in vivo*, we first tested a catecholamine challenge with isoproterenol as a single agent using a dose (3 mg/kg) that reliably induces ventricular arrhythmias in Casq2^−/−^ mice (Watanabe et al., 2009; Katz et al., 2010). The same isoproterenol dose induced only a few PVC’s in 1 out of 7 RyR2^R4496C+/–^ mice, which makes it difficult to test flecainide efficacy. Hence, we tested a more intense catecholamine challenge by including the RyR2 activator caffeine (Kong et al., 2008). After challenge with isoproterenol plus low dose caffeine (60 mg/kg; LOW), 100% of Casq2^−/−^ mice had PVC’s. In contrast, only 29% of RyR2^R4496C+/–^ mice exhibited PVCs (77.8 ± 34.7 vs. 11.8 ± 6.6 PVC/min, *p* < 0.05; Figures 4B,C). Ventricular tachycardia (VT) was observed in 87.5% of Casq2^−/−^ mice, but not in RyR2^R4496C+/–^ mice. Next, we tested a higher dose of caffeine (120 mg/kg; HIGH) in combination with isoproterenol. The incidence of ventricular arrhythmias after high-dose challenge increased to 75% in RyR2R^4496C+/–^ mice. Taken together, these results indicate that the Casq2^−/−^ mice have a more severe CPVT phenotype compared to RyR2^R4496C+/–^ mice, which mirrors the results from the single cell studies (Figures 2, 4).

Next, we tested the efficacy of flecainide *in vivo* (Figure 4). Flecainide was highly effective in both models challenged with low dose caffeine and isoproterenol. Pretreatment with flecainide completely prevented PVCs in RyR2^R4496C+/–^ mice and drastically reduced the incidence of PVCs in Casq2^−/−^ mice (Figure 4). The protective effect of flecainide against CPVT was less pronounced in mice challenged with the highest caffeine dose (Figure 4). Taken together, the results indicate that flecainide efficacy in CPVT is *mutation-independent* both *in vitro* and *in vivo*.

## Discussion

The main finding of the present work is that flecainide has comparable efficacy in suppressing spontaneous Ca^2+^ waves and ventricular arrhythmias in both the Casq2^−/−^ and the RyR2^R4496C+/–^ CPVT models. Flecainide’s mutation-independent efficacy in the CPVT animal models is consistent with its clinical efficacy in patients with RyR2 and Casq2 mutations (van der Werf et al., 2011). We also observe flecainide has a relatively slow onset of action in isolated cardiomyocytes (maximum effect after 30 min) and that flecainide’s antiarrhythmic effect is reduced in experimental conditions such as Ca^2+^ overload in single cells or high-dose catecholamine challenge *in vivo*, which may have contributed to some of the divergent experimental results in the literature.

Catecholaminergic polymorphic ventricular tachycardia is one of the most severe genetic ventricular arrhythmia and sudden death syndromes, and the search for options to treat CPVT patients has challenged physicians and researchers in cardiovascular pharmacology. Since the initial reports, the use of ß-blockers has shown good therapeutic efficacy in CPVT patients (Leenhardt et al., 1995; Lahat et al., 2001). Ca^2+^ channel blockers have been suggested as addition to ß-blockers (Katz et al., 2010; Pflaumer and Davis, 2012). However, pharmacological therapy with ß-blockers and Ca^2+^ channel blockers are not completely effective in up to 50% of CPVT patients (Pflaumer and Davis, 2012). The class Ic anti-arrhythmic drug flecainide, commonly known as an inhibitor of membrane Na^+^ channels, has been shown to have clinical efficacy for preventing ventricular arrhythmias in CPVT patients carrying either Casq2 or RyR2 mutations (Watanabe et al., 2009). Also, flecainide reduced arrhythmias in patients resistant to standard pharmacological therapy (van der Werf et al., 2011). Flecainide suppressed ventricular arrhythmias and inappropriate ICD shocks even in the presence of ß-blockers (Hong et al., 2012; Jacquemart et al., 2012), which was recently confirmed in a randomized clinical trial (Kannankeril et al., 2017). Despite its proven clinical efficacy in CPVT, the mechanism of flecainide action remains controversial. The purpose of the current study was to test whether the different results in the literature can be partly explained by different CPVT mouse models and experimental protocols used for testing the effects of flecainide.

We find that Casq2^−/−^ mice have a stronger pro-arrhythmic response to β-adrenergic stimuli compared to RyR2^R4496C+/–^ mice. These data agree with previous reports that catecholamine challenge with isoproterenol as a single agent is sufficient to induce ventricular arrhythmias in Casq2^−/−^ mice (Watanabe et al., 2009; Katz et al., 2010). Liu et al. (2011) reported an incidence of VT of 70% in RyR2^R4496C+/–^ mice by using a mixture of epinephrine (2 mg/kg) and caffeine (120 mg/kg). However, in Casq2^−/−^ mice, a much smaller dose of epinephrine (0.5 mg/kg) as a single agent was sufficient to induce PVC’s and non-sustained VT in 100% of mice tested (Katz et al., 2010). Caffeine is known to increase SR Ca^2+^ release by direct binding to RyR2. Hence, we used a catecholamine challenge of isoproterenol plus caffeine (low and high dose) to achieve a robust level of arrhythmias in both animal models. Flecainide completely suppressed PVCs in RyR2^R4496C+/–^ and strongly reduced PVCs in Casq2^−/−^ mice. These findings further support the hypothesis that flecainide is effective in reducing arrhythmias independent of the causative mutation (Watanabe et al., 2009; Liu et al., 2011).

Consistent with the more severe CPVT phenotype *in vivo*, ventricular myocytes from Casq2^−/−^ mice showed a higher incidence of spontaneous Ca^2+^ waves compared to RyR2^R4496C+/–^ cells (Figures 2, 3). This result matches published values for the high incidence of Ca^2+^ waves in Casq2^−/−^ cardiomyocytes (Watanabe et al., 2009; Hwang et al., 2011), whereas the incidence was lower in RyR2^R4496C+/–^ cells as reported by Liu et al. (2011). Interestingly, Liu et al. (2011) failed to find flecainide efficacy against Ca^2+^ waves in cardiomyocytes isolated from RyR2^R4496C+/–^ mice. Our data clearly show that flecainide is effective against Ca^2+^ waves in cardiomyocytes from RyR2^R4496C+/–^ mice, unless the cardiomyocytes are Ca^2+^ overloaded (Figure 3). We note that the frequency of Ca^2+^ sparks reported by Liu et al. (2011) was very high, which may indicate the presence of high cytosolic [Ca^2+^] consistent with Ca^2+^ overload. Furthermore, our group reports SCR incidence of 5% in isolated RyR2^R4496C+/–^ cells, while Liu et al. report an incidence of 87% at an extracellular [Ca^2+^] of 2 mM. We observe such high rates only in [Ca^2+^] overload conditions, further highlighting the discrepancy in experimental conditions. Our data reported here show that flecainide’s inhibitory effect on Ca^2+^ waves decreases as intracellular Ca^2+^ increases regardless of the underlying CPVT mutation. As we previously reported (Hilliard et al., 2010; Savio-Galimberti and Knollmann, 2015), flecainide inhibits Ca^2+^ waves by reducing Ca^2+^ release from individual RyR2 channel clusters known as Ca^2+^ sparks, as evidenced by the reduced Ca^2+^ spark mass. Note that there is a compensatory increase in Ca^2+^ spark frequency after flecainide application. As a result, flecainide has no effect on SR Ca^2+^ leak and SR Ca^2+^ content (Hilliard et al., 2010; Savio-Galimberti and Knollmann, 2015). Since increasing cytosolic Ca^2+^ activates RyR2 channels, more RyR2 clusters will fire spontaneously. Although not directly tested here, we postulated that the increased activity of neighboring RyR2 clusters renders the block of individual RyR2 clusters by flecainide increasingly ineffective as diastolic Ca^2+^ increases to high levels.

Our group has attributed flecainide’s striking efficacy against spontaneous Ca^2+^ waves *in vitro* and antiarrhythmic efficacy *in vivo* to dual Na^+^ and RyR2 channel inhibition (Watanabe et al., 2009; Hilliard et al., 2010; Galimberti and Knollmann, 2011; Hwang et al., 2011). Although disputed by Bannister et al. (2015) the most plausible explanation is that flecainide acts directly on RyR2 by blocking its open state, and thereby reduces the amount of Ca^2+^ released during spontaneous openings of RyR2 channel clusters measured as Ca^2+^ sparks (Hilliard et al., 2010). The hypothesis that flecainide inhibits RyR2 channels is supported by work indicating that flecainide suppresses RyR2-mediated SR Ca^2+^ efflux (e.g., Ca^2+^ spark mass) in permeabilized cardiomyocytes under experimental conditions that render Na^+^ channels inactivated (Hilliard et al., 2010; Savio-Galimberti and Knollmann, 2015; Batiste et al., 2019). In contrast, Sikkel et al. (2013) proposed that Na^+^ channel block alone is responsible for Flecainide’s efficacy in CPVT. We note that Sikkel et al. applied flecainide only for 5 min in the experiments comparing different Na^+^ channel blockers to test the contribution of RyR2 block on Ca^2+^ sparks and waves. We find that flecainide has to be applied for more than 10 min to single cells in order to inhibit Ca^2+^ waves (Figure 1). Hence, differences in experimental conditions (i.e., incubation time, the degree of Ca^2+^ overload) may have contributed to the lack of flecainide activity on Ca^2+^ waves in some reports.

## Data Availability

All datasets generated for this study are included in the manuscript and/or the supplementary files.

## Ethics Statement

All protocols were following the Guide for the Care and Use of Laboratory Animals (NIH publication No. 85-23, revised 1996) and approved by the Vanderbilt University Institutional Committee of Ethics in Animal Research.

## Author Contributions

HH conducted and analyzed the isolated myocyte experiments. MB and MF conducted and analyzed the *in vivo* mouse ECG experiments. MB and HH wrote the initial draft of the manuscript. JR re-analyzed ECG recording and prepared the figures. HH and BK designed and supervised the overall project, and prepared the final manuscript and figures.

## Conflict of Interest Statement

The authors declare that the research was conducted in the absence of any commercial or financial relationships that could be construed as a potential conflict of interest.
